# Ethical Challenges of Artificial Intelligence in Medicine

**DOI:** 10.7759/cureus.74495

**Published:** 2024-11-26

**Authors:** Ava L Boudi, Max Boudi, Connie Chan, F Brian Boudi

**Affiliations:** 1 Biochemistry, Arizona State University, Tempe, USA; 2 Education, Arizona State University, Phoenix, USA; 3 Internal Medicine, University of Arizona College of Medicine, Phoenix, USA; 4 Cardiology, University of Arizona College of Medicine, Phoenix, USA

**Keywords:** artificial intelligence in radiology, clinical genetics ethics, ethic consideration, ethics in ai, meditation and genetics

## Abstract

Artificial intelligence (AI) and machine learning (ML) have become critical components in the transformation of healthcare. They offer enhanced diagnostic accuracy, personalized treatment plans, and support for clinical decision-making. However, with these advancements come significant ethical challenges, including concerns around transparency, bias, data privacy, and the potential displacement of healthcare professionals. This review delves into these ethical concerns, issues of transparency, data privacy, bias, and the moral responsibility of decision-making with a particular focus on the role of AI in new drug/genetic treatment discovery, exploring how AI models are employed in protein, RNA, and DNA structural prediction to accelerate drug development. Addressing these challenges is crucial for ensuring that AI is used responsibly, benefiting patients while maintaining trust in the healthcare system.

## Introduction and background

Artificial intelligence (AI) has revolutionized various sectors, including healthcare. Machine learning (ML), a subset of AI, enables systems to process vast datasets and recognize patterns that often go undetected by human clinicians. AI refers to computational methods designed to simulate human intelligence, while ML focuses specifically on training algorithms to learn from data and improve predictions over time. AI has been pivotal in improving diagnostic accuracy, personalizing treatments, and supporting clinical decision-making. Beyond drug discovery, AI is transforming other areas of healthcare, including radiology, where it enhances diagnostic accuracy for conditions like cancer and cardiovascular diseases through advanced imaging analysis. In gastroenterology, AI aids in the detection of polyps during colonoscopy and improves the diagnosis of gastrointestinal disorders. In the same way, in pathology, AI-driven tools are expediting the analysis of tissue samples, identifying abnormalities with precision, and supporting more accurate diagnoses. These diverse applications highlight the expansive scope of AI in modern medicine, improving patient care across multiple specialties [1]. However, as AI continues to evolve, it raises significant ethical questions about transparency, bias, and accountability in medicine [2].

The increasing significance of AI in drug discovery has introduced both new possibilities and challenges. This advancement enables a more rapid and efficient identification of potential drug candidates, significantly reducing the time and costs involved in the development process. It allows researchers to explore novel therapeutic targets and may lead to the creation of personalized medications tailored to individual patients, thereby improving the likelihood of successful drug approvals. AI algorithms are expediting the discovery of neoplasms and therapeutic targets through the analysis of protein, RNA, and DNA structures. However, as AI models become more integrated into the drug development pipeline, concerns regarding data privacy, model transparency, and the ethical ramifications of decision-making without adequate human oversight and accountability continue to be pressing issues [3,4].

AI systems pose significant challenges to data protection and privacy due to the vast amounts of personal data they process. Without adequate safeguards, these systems can lead to unauthorized access, re-identification of individuals, biased outcomes, and a lack of transparency, resulting in privacy breaches and reputational harm. Key risks include excessive data collection without consent, data breaches exposing sensitive information, the re-identification of anonymized data, secondary uses of data without permission, biased algorithmic outcomes, opaque decision-making processes, surveillance through facial recognition or location tracking, and the creation of "deepfakes" and misinformation [5].

Mitigating these risks requires a proactive approach, including data minimization to limit collection to what is strictly necessary, obtaining informed consent through clear communication, anonymizing personal data where feasible, embedding privacy considerations into AI design (“privacy-by-design”), ensuring transparency in AI decision-making, and regularly monitoring data practices and models for emerging risks [5].

This review addresses the potential of AI in drug discovery and the ethical dilemmas it introduces, emphasizing the importance of implementing robust ethical frameworks to guide AI’s use in healthcare [3,6].

## Review

As shown in Table 1, the applications of AI in medicine offer immense potential but are accompanied by complex ethical challenges that require proactive strategies to address risks and ensure equitable implementation. 

**Table 1:** Summary of Ethical Challenges and Applications of AI in Medicine SHAP: SHapley Additive eXplanations; LIME: Local Interpretable Model-agnostic Explanations; FIRM: Fairness in Machine Learning.

Aspect	Details	Examples				
Applications in Medicine	Diagnostics, personalized treatments, drug discovery, radiology, pathology, and GI disorder detection.	AI in colonoscopy for polyp detection.				
AI in Drug Discovery	Protein structure prediction, RNA/DNA folding analysis, small-molecule virtual screening.	AlphaFold, AtomNet, Schrödinger platforms.				
Ethical Challenges	Data privacy, bias, transparency, accountability, and workforce displacement.	Genomic data breaches, biased datasets.				
Key Risks	Data breaches, re-identification of anonymized data, biased outcomes, opaque decision-making.	23andMe breach, algorithm bias in oncology.				
Mitigation Strategies	Privacy-by-design, federated learning, diverse datasets, explainable AI, dynamic ethical oversight.	WHO 2023 Ethical Guidelines.				
Validation	Experimental techniques to confirm AI predictions.	X-ray crystallography, NMR spectroscopy.				
Workforce Implications	Job displacement concerns; opportunities in algorithm development and clinical trial optimization.	AI in radiology; reskilling initiatives.				
Transparency Techniques	Enhancing interpretability of AI models with SHAP, LIME, and attention mechanisms.	Visual explanations for medical imaging.				
Accountability Frameworks	Shared responsibility among developers, providers, and institutions.	Fairness in Machine Learning (FIRM).				

AI and ML in drug discovery

One of the most promising applications of AI in healthcare is drug discovery. Traditional drug discovery is a lengthy and costly process, with estimates suggesting it can take over 10 years and billions of dollars to bring a single drug to market [7]. AI offers the potential to shorten this timeline significantly by identifying promising drug candidates early in the development process.

AI in protein structure prediction

AI’s role in predicting protein structures has been one of the most exciting advancements in recent years. Proteins are essential biological molecules, and their 3D structures largely determine their functions. Accurately predicting these structures is crucial for designing effective drugs that can target specific proteins involved in diseases.

DeepMind’s AlphaFold, a revolutionary AI model, has achieved remarkable accuracy in predicting protein structures, outperforming traditional experimental methods like X-ray crystallography and NMR spectroscopy [4]. This has accelerated drug discovery efforts, as researchers can now model the structure of disease-related proteins and design drugs that interact with them more precisely. AlphaFold has been used to predict the structures of proteins implicated in neurodegenerative diseases, cancer, and infectious diseases, offering new insights into drug design [8]. AlphaFold's predictions depend heavily on the training datasets, which are derived from existing protein databases. These datasets may lack representation of rare or novel proteins, reducing the model's ability to generalize and predict accurately across diverse biological contexts. AlphaFold is highly effective for static protein structures but is less accurate when predicting dynamic interactions, such as those seen in multimeric protein complexes or transient conformational states. This limitation is particularly evident in modeling interactions influenced by unique cellular environments. Real-world protein behavior often depends on environmental conditions like pH, ionic strength, and cellular compartments. AlphaFold does not fully account for these factors, which limits its applicability in predicting functionality-relevant conformations in vivo. 

However, the use of AI in protein structure prediction raises ethical concerns, particularly regarding data bias and the possible use of such data by insurance companies to deny coverage to high-risk individuals. AI models must be trained on diverse datasets to ensure they perform well across a wide range of proteins. If AI models are trained primarily on proteins from certain species or disease types, they may not generalize well to others, potentially leading to biased outcomes in drug discovery [9].

AI in RNA and DNA structure prediction

RNA and DNA play critical roles in gene expression and disease development, and understanding their structures is key to developing targeted therapies. AI has shown great potential in predicting the folding patterns of RNA and DNA, which can influence gene regulation and protein synthesis. AI models are being used to predict how changes in DNA structure can lead to diseases like cancer, guiding the development of gene therapies and personalized medicine [10].

AI’s role in RNA-based therapies, such as those used in COVID-19 mRNA vaccines, is particularly noteworthy. By predicting the structure of RNA molecules, AI can help design more effective vaccines and treatments for infectious diseases. However, ethical challenges arise in the use of genomic data to train these AI models. Genomic data is highly personal, and there are concerns about data privacy, particularly in light of recent high-profile breaches of healthcare data [11]. In October 2023, genetic testing company 23andMe experienced a data breach of 6.9 million users where personal information and ancestry details and, some health-related data, primarily through the "DNA Relatives" feature were compromised [12]. Ensuring the responsible and secure use of genomic data is crucial to maintaining patient trust and protecting sensitive health information.

AI in small-molecule drug discovery

AI’s capabilities extend beyond biological macromolecules to include the discovery of small-molecule drugs. AI can screen vast libraries of chemical compounds to identify those with the highest likelihood of interacting with disease-related proteins. This process, known as “virtual screening”, dramatically reduces the time required to identify lead compounds for drug development [13].

Recent AI platforms, such as AtomNet and Schrodinger’s Drug Discovery Suite, have successfully identified novel drug candidates by predicting how small molecules bind to protein targets [14,15]. These platforms use deep learning models to simulate protein-ligand interactions, predicting the efficacy of potential drug candidates with unprecedented accuracy.

However, as with protein and RNA prediction, there are ethical concerns surrounding AI’s use in small-molecule drug discovery. The data used to train AI models may be biased towards certain chemical libraries or drug targets, potentially limiting the diversity of drugs that can be discovered. Furthermore, the “black box” nature of many AI models raises concerns about transparency, as researchers and clinicians may not fully understand how AI systems arrive at their conclusions [16].

Validation techniques for AI predictions

Validation against experimental methods such as X-ray crystallography, cryo-electron microscopy, and nuclear magnetic resonance (NMR) spectroscopy is essential. These methods provide high-resolution data that can confirm or refine AI-generated structures, ensuring that predictions are biologically relevant and reliable [3]. Structural inaccuracies can have far-reaching consequences, especially in drug development. Misidentified binding sites or poorly predicted protein conformations can lead to ineffective or unsafe drug candidates. Therefore, iterative validation with experimental feedback is essential for mitigating errors and ensuring robust applications in therapeutic contexts. By addressing these challenges and leveraging experimental validation, the reliability and applicability of AI-driven structural predictions can be further enhanced.

Ethical challenges in AI-driven healthcare

Data Privacy and Security

AI systems rely on vast amounts of data to function effectively. In healthcare, this often includes sensitive patient data, including genomic information, medical histories, and clinical records. Protecting this data is essential to maintaining patient trust and preventing unauthorized access or misuse. AI-driven healthcare tools are vulnerable to cyberattacks, and recent breaches in major hospital systems have highlighted the need for robust security measures including breaches, such as the 23andMe hack, that demonstrate the vulnerabilities in genomic data protection [17]. 

Moreover, the use of patient data in training AI models raises concerns about informed consent and data ownership. Patients may need to fully understand how their data is being used or may not have provided explicit consent for its use in AI-driven research. Ensuring that AI systems comply with data privacy regulations, such as the General Data Protection Regulation (GDPR), HIPAA (USA), GDPR (EU), and PIPEDA (Canada) is critical to addressing these ethical concerns and maintaining patient trust [18].

Bias in AI Models

Bias in AI models is a significant concern, particularly in healthcare. AI systems trained on biased or non-representative data may produce biased outcomes, leading to disparities in diagnosis, health equity [19], treatment, and drug discovery. The integration of AI into healthcare must address systemic disparities. Recent advancements demonstrate how inclusive datasets and public-private partnerships can reduce algorithmic bias, enhancing health equity.

To mitigate these risks, AI models will need to be trained on diverse and representative datasets. Regular audits and algorithmic transparency will also be necessary to identify and correct biases before they cause harm. In the context of drug discovery, ensuring that AI models are trained on diverse chemical and biological datasets can help prevent biased outcomes in the development of new treatments [20].

Real-world examples and studies

Studies have shown that AI models trained predominantly on lighter skin tones result in lower accuracy in diagnosing dermatological conditions for darker-skinned patients. For example, AI models used in dermatology have been shown to perform poorly on patients with darker skin tones, exacerbating existing healthcare inequalities [21]. This can delay or misdirect treatment for certain populations. An algorithm used to allocate healthcare resources in the United States was found to underestimate the health needs of Black patients due to biased training data that relied heavily on historical healthcare spending rather than actual medical needs [22]. 

Efforts should focus on collecting and curating datasets that represent a wide range of demographics, ensuring inclusivity across gender, ethnicity, and socioeconomic factors. Conducting bias audits of AI models can identify and address disparities before deployment. This includes testing models on various subpopulations to measure performance equity. Publishing model architectures and datasets allow external parties to evaluate and challenge potential biases. 

Practical implementation of ethical frameworks

To ensure successful adoption, ethical frameworks must address real-world complexities. Recent developments have focused on Dynamic Ethical Oversight. Institutions like the World Health Organization (WHO) are advocating for adaptable ethical guidelines that evolve with advancements in AI, ensuring continued relevance [23]. Federated learning model studies demonstrate how federated learning enables AI training across decentralized datasets without compromising patient privacy, aligning with ethical data governance principles [24].

Integrating these strategies with interdisciplinary stakeholder input ensures that ethical AI solutions remain practical and effective.

Techniques for interpretability

SHapley Additive exPlanations (SHAP) values assign importance scores to input features, helping clinicians understand why an AI model makes specific predictions. This is particularly valuable for understanding risk scores or diagnostic recommendations. Local Interpretable Model-agnostic Explanations (LIME) provides a simplified explanation of model predictions by approximating them locally with interpretable models. It has been applied to diagnostic imaging and patient stratification in healthcare. Attention Mechanisms, in neural networks, attention maps highlight the areas of input data (e.g., regions in medical images) that influence the output, offering visual insights to support clinical decision-making. 

Transparency and the black box problem

AI systems and intense learning models often operate as “black boxes” where the decision-making process is opaque. In healthcare, this lack of transparency can be problematic, as clinicians need to understand how AI systems arrive at their recommendations to ensure patient safety. The black box problem is especially concerning in drug discovery, where AI predictions about the efficacy of potential drug candidates must be thoroughly vetted to avoid costly and harmful errors [25].

Recent advancements in explainable AI (XAI) are helping to address this issue by making AI models more transparent and interpretable. However, there still needs to be a significant gap between the current capabilities of AI systems and the level of transparency required for healthcare applications. More work is needed to ensure that AI systems in drug discovery are both accurate and explainable, allowing researchers and clinicians to maintain control over critical decisions [26].

Accountability in decision-making

As AI systems take on more decision-making responsibilities in healthcare, questions about accountability become increasingly important. Sometimes, AI-generated outcome summaries contain errors, known as hallucinations and omissions. If an AI system makes a mistake-whether in diagnosing a patient or recommending a drug candidate-who bears responsibility? The developers of the AI system, the pharmaceutical company, or the healthcare providers who implemented the system? This question highlights the importance of frameworks like Fairness in Machine Learning (FIRM), which emphasize transparency, equity, and clear oversight mechanisms.

Current legal frameworks generally hold healthcare professionals accountable for clinical decisions, even when AI is involved. However, as AI systems become more autonomous, this model may need to be reconsidered. A shared accountability model, where responsibility is distributed between AI developers, healthcare providers, and institutions, may provide a more appropriate framework for AI-driven decision-making [27].

Workforce displacement and job restructuring

AI’s growing role in healthcare raises concerns about workforce displacement, particularly in diagnostic fields like radiology and pathology, where AI can analyze medical images and data at speeds far exceeding human capabilities [28]. However, AI also presents opportunities to augment human expertise rather than replace it. In drug discovery, AI can assist researchers by analyzing large datasets and identifying patterns that may not be immediately apparent to humans, allowing them to focus on more complex and creative aspects of drug development [29].

The ethical challenge is ensuring that AI is used to complement human expertise rather than replace it. Healthcare institutions and pharmaceutical companies must integrate AI in a way that supports healthcare professionals and enhances their roles, rather than rendering them obsolete [30]. 

AI-driven advancements may create roles in the following:

Algorithm Development Specialists

Clinicians with AI expertise can contribute to tailoring models for specific clinical contexts, bridging the gap between developers and end-users.

Clinical Trial Optimization

AI can streamline participant selection and trial monitoring, creating roles focused on integrating AI outputs into trial design and execution.

To utilize these opportunities, healthcare institutions must actively invest in workforce reskilling programs, supported by academic partnerships and government initiatives. For instance, initiatives like the “AI in Healthcare Workforce Alliance” provide tailored training modules to address this transition.

## Conclusions

AI is redefining the boundaries of healthcare, spearheading innovations in diagnostics, treatment personalization, and drug discovery. Its ability to decode the complexities of protein, RNA, and DNA structures is unlocking solutions to previously intractable medical challenges. Yet, as AI reshapes medicine, it brings ethical challenges that demand urgent attention. A future where AI responsibly drives healthcare innovation is achievable only through robust ethical frameworks, interdisciplinary collaboration, and a commitment to equity and transparency. By addressing these concerns proactively, we can ensure AI not only transforms medicine but does so with integrity, trust, and accountability.
